# Limited genetic diversity found among genotypes of the Entada landrace (*Ensete ventricosum*, (Welw.) Chessman) from Ethiopia

**DOI:** 10.3389/fpls.2024.1336461

**Published:** 2024-09-09

**Authors:** Alye Tefera Haile, Mallikarjuna Rao Kovi, Sylvia Sagen Johnsen, Trine Hvoslef-Eide, Bizuayehu Tesfaye, Odd Arne Rognli

**Affiliations:** ^1^ Faculty of Biosciences, Department of Plant Sciences, Norwegian University of Life Sciences (NMBU), Ås, Norway; ^2^ School of Plant and Horticultural Science, College of Agriculture, Hawassa University, Hawassa, Ethiopia

**Keywords:** enset, SNP markers, ddRAD, outlier SNPs, genetic diversity

## Abstract

The Entada landrace of enset (*Ensete ventricosum* (Welw.) Chessman) is probably the most unique indigenous crop in Ethiopia, being maintained and utilized by the Ari people in the South of Ethiopia. Here we describe genetic diversity, selection signatures and relationship of Entada with cultivated and wild enset using 117 Entada genotypes collected from three Entada growing regions in Ethiopia (Sidama, South and North Ari). A total number of 1,617 high-quality SNP markers, obtained from ddRAD-sequences, were used for the diversity studies. Phylogenetic analysis detected a clear distinction between cultivated enset, Entada and wild enset with Entada forming a completely separated clade. However, extremely short branch lengths among the Entada genotypes indicate very little molecular evolution in the Entada lineages. Observed and expected heterozygosities were high, 0.73 and 0.50, respectively. Overall, our results strongly indicate that the Entada genotypes we have studied originated from one or a few clonal lineages that have been propagated and spread among farmers as clones. Prolonged clonal propagation of heterozygous genotypes from a single or few founding lineages has led to populations with very little or no diversity between genotypes, and high heterozygosity within genotypes. Signatures of directional selection were identified at eight loci based on an F_ST_ outlier analysis. Four candidate genes detected are involved in axillary shoot growth and might be involved in controlling natural sucker formation in Entada.

## Introduction

Landraces are geographically and ecologically distinctive populations (Brown, 1978), which are highly diverse containing a mixture of genotypes (Hawkes, 1983). They often have comparative advantages over commercial cultivars because they have been selected to survive stressful conditions and can be cultivated using low input and/or organic cultivation methods. Moreover, there is an increasing interest in finding new food sources to alleviate malnutrition. The local landraces constitute valuable germplasm for plant breeding, and it is important to conserve the genetic diversity present in landraces (Kölliker et al., 2003).

Most of the plant landraces are cultivated and maintained by smallholder farmers and private gardeners all over the world. Numerous landraces have originated as a result of agriculture and horticulture over the past 10,000 years (Zeven, 1998). Landraces are commonly considered as locally adapted and endemic to a specific area. Many of the plant species that are cultivated for food are neglected and underutilized despite their crucial role in the food security, nutrition, and income generation of rural societies (Magbagbeola et al., 2010). Rural communities prepare food and different products such as medicine, shelter, feed, and fuel from different orphan crops. Mostly, underutilized crops make up a significant part of the diet of rural households, typically during periods of drought, famine, and dry seasons (Campbe, 1987).

**Figure 1:**
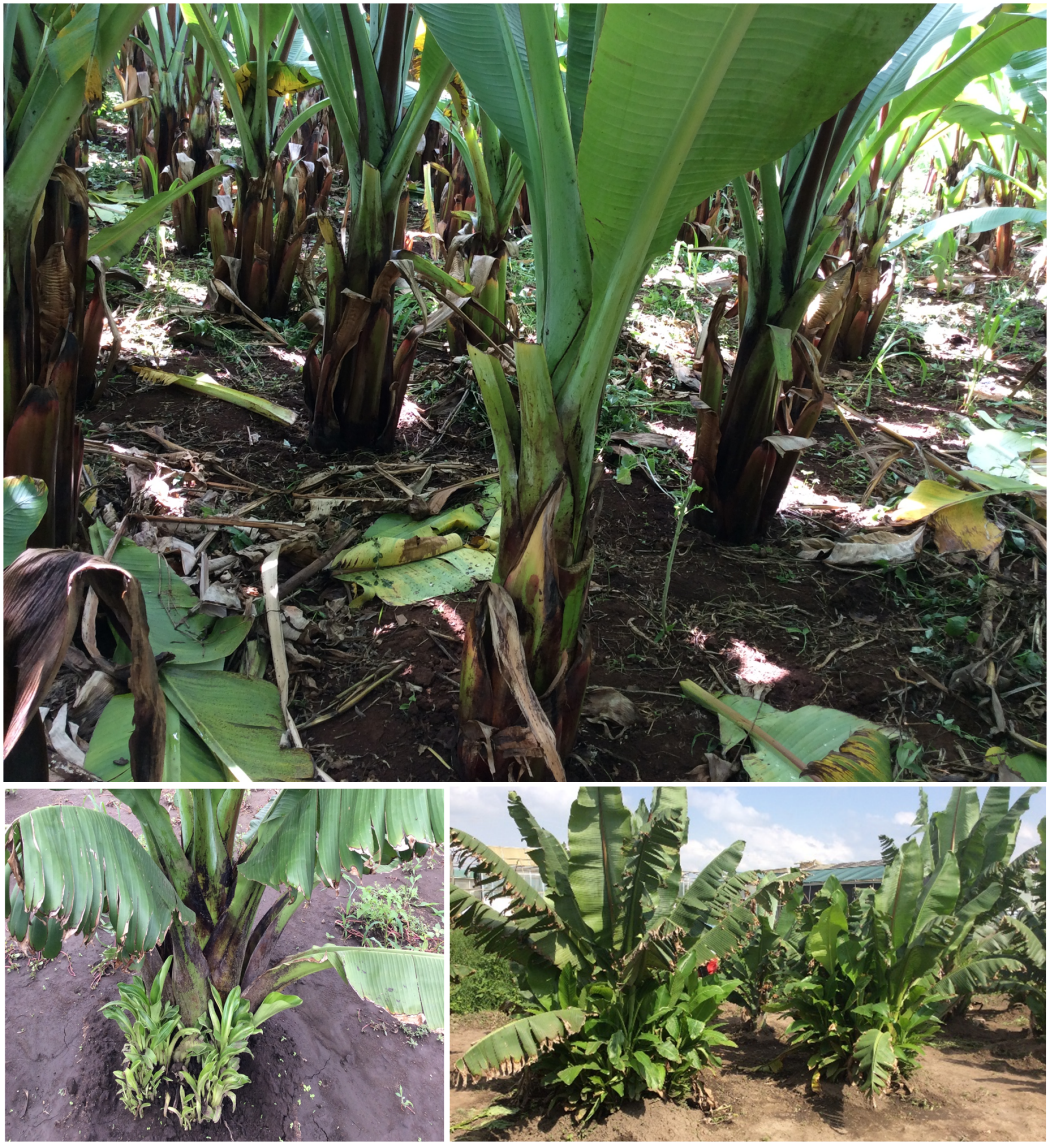
Cultivated enset (above) and Entada with natural suckers (below). Photos by A.T.Haile.

In Ethiopia, the Entada landrace of enset (*Ensete ventricosum* (Welw.) Cheesman) is probably the most unique and understudied indigenous landrace. Entada is an enset landrace that has lost apical dominance present in enset; therefore, it is propagated by natural suckers like banana (Shigeta, 1990). The primary distinction between the cultivation of enset and Entada lies in the growth of suckers. Entada generates suckers naturally, while they need to be induced in enset (**Figure 1**). The local or farmers name “Intada” indicates that the plant grows or multiplies by itself (Shigeta, 1992). Entada rarely flower and set fruits, and it is different morphologically from all other cultivated and wild ensets, which are not propagated by suckers (Olango et al., 2015; Shigeta, 1992). Genotypes of Entada are being maintained and utilized mainly by the Ari people in the Southern regions of Ethiopia (Shigeta, 1992). The major processed foods from Entada are Amicho prepared from the underground corm (the underground base of the stem that serves as a storage organ). The fresh corm is cooked like potato and yam. It is a multi-purpose crop with cultural values in religious ceremonies and as a feed source in addition to being a food. Due to its tolerance to adverse factors such as drought and different soil types, this landrace is considered an alternative crop for areas with extreme growing conditions (Brandt et al., 1997; Shigeta, 1992). However, so far there are no genetic diversity studies among and within Entada landrace (Olango et al., 2015; Shigeta, 1992).

A previous molecular diversity study of enset using SSR (microsatellites) and SNP (single nucleotide polymorphism) markers revealed that Entada belongs to the genus *Ensete* of the Musaceae family (Olango et al., 2015; Yemataw et al., 2018). Currently, several new molecular techniques are being applied together with phenotypic descriptions to investigate genetic diversity and relatedness in enset accessions (Birmeta et al., 2004). Single nucleotide polymorphisms (SNPs) and simple sequence repeats (SSRs) are the most common DNA markers for genetic diversity studies (Tsykun et al., 2017). Among all DNA markers, SNPs are abundant and robust markers, which are well suited for automated high-throughput genotyping of large numbers of samples. Besides, SNPs are able to resolve the differences among extremely similar individuals and increase the accuracy of diversity estimates (Hinze et al., 2017). A recent study applied tGBS (tunable genotyping-by-sequencing) derived SNP markers to study domestication of enset using 192 farmer-identified enset landraces, 14 wild and 7 semi-domesticated ensets (White et al., 2023).

Double digest restriction-site associated DNA (ddRADseq) is one of the reduced representation sequencing methods for SNP discovery at a genome-wide scale. It includes digesting the DNA with two restriction enzymes to allow greater control of the genomic regions sampled for sequencing and more reproducible recovery of sequenced regions. Specific size-selected fragments are generated and sequenced (Peterson et al., 2012). ddRADseq eliminates the random shearing step of the original RAD protocol (Baird et al., 2008). The application of ddRADseq has been successfully applied in many plant species such as tomato (Esposito et al., 2020), strawberry (Davik et al., 2015), northern red oak (Konar et al., 2017), and oriental thuja (*Platycladus orientalis*) (Jin et al., 2019). Here, we report on the development and utilization of the first set of SNP markers developed from ddRAD sequences to study genetic diversity and relatedness among Entada genotypes, cultivated and wild enset collected from the Entada growing regions in Ethiopia. To facilitate progress in future Entada conservation and breeding, it is essential to discover and characterize Entada and understand its relationships to cultivated and wild enset populations.

In the present study we applied SNP markers to: (1) study genetic diversity and relationship of Entada genotypes with cultivated and wild enset, (2) determine the effects of clonal propagation on genetic diversity, and (3) identify candidate genes involved in sucker formation in Entada.

## Materials and methods

### Plant samples and treatments

**Figure 2:**
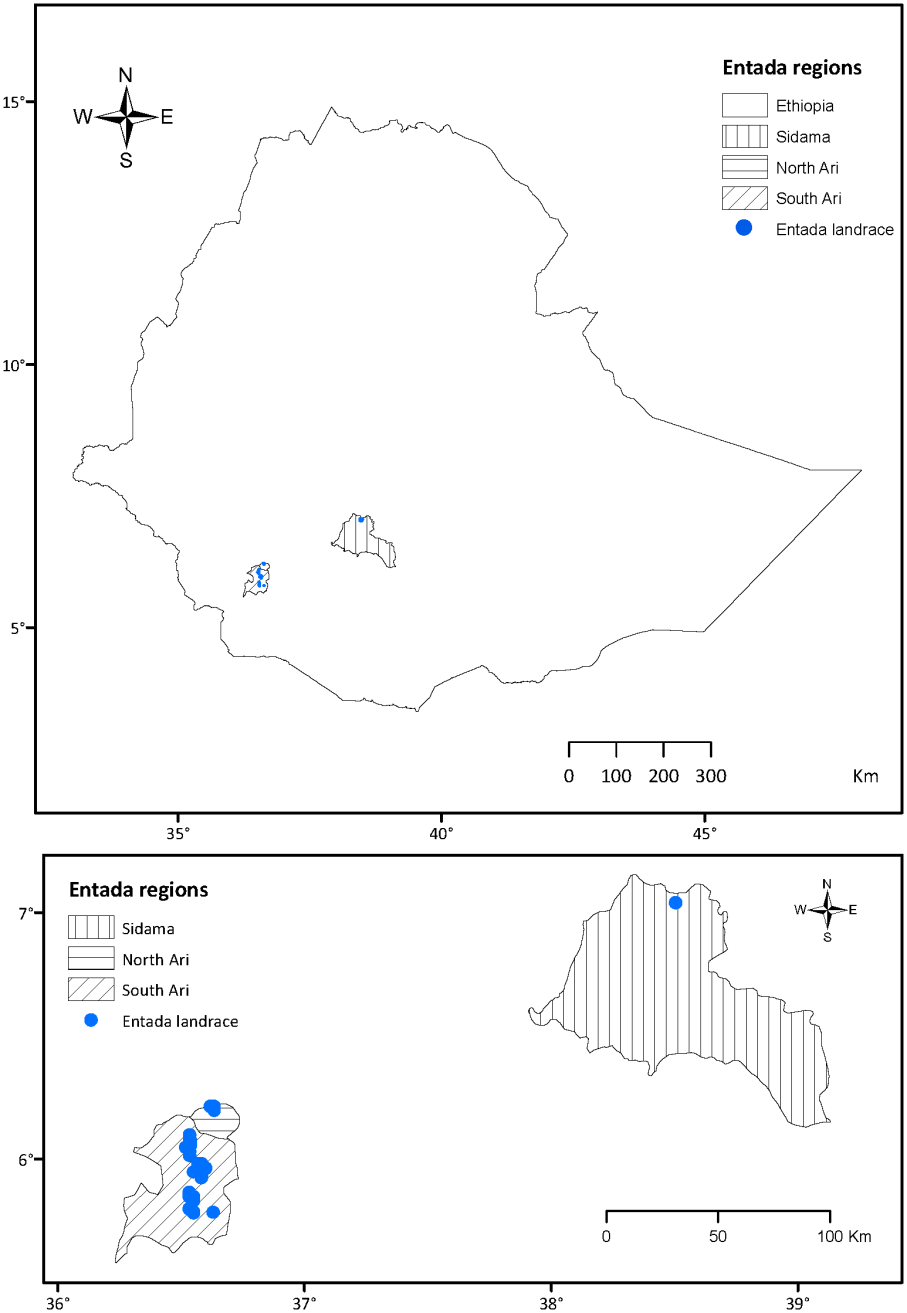
Geographic origin of the Entada genotypes collected in the Sidama, North Ari and South Ari regions of Ethiopia.

Leaf tissue from 129 Entada individuals were collected from the South Ari (91), North Ari (17) and Sidama (21 from the germplasm collection at Hawassa University) regions in Ethiopia (**Figure 2**; **Supplementary Table 1**). The saturated NaCl-CTAB solution was used to preserve the Entada leaf samples upon collection, as described by Rogstad (1992), with minor modifications. Briefly, 550 g NaCl was added to 1 L of water, boiled, and cooled at ambient temperature, and mixed thoroughly until the salt precipitated. Then, 35 g of CTAB was added gradually with intermittent irregular intervals mixing, until the solution became viscous. 35-40 mL of the prepared solution was aliquoted into 50 mL Falcon tubes and used for preservation of tissue samples. A pair of scissors was used to remove leaf samples from the mother plants, and the scissors were cleaned with ethanol (96%) between independent samples. Fresh cigar-leaf samples harvested from each genotype were stored immediately in the 50 mL tubes containing the saturated NaCl-CTAB preservation buffer. Samples were then placed in a black plastic bag and stored in a dark room at ambient temperature, to preserve genomic DNA from degradation during transportation from the farmer fields in Ethiopia to the laboratory in Norway. Upon arrival at the laboratory, the saturated NaCl-CTAB solution was washed off thoroughly with deionized water and excess water wiped off the leaves with dry white wipes (Kimberly-Clark™ Professional Kimtech Science™). Leaf samples were put in liquid nitrogen, ground quickly using a pestle and mortar, and the ground powder transferred into 2 mL microcentrifuge tubes (Eppendorf A.G., Hamburg, Germany). Pestles and mortars were washed and dried before starting each sample preparation, and all pulverized leaf samples stored at -80°C until further analyses. For DNA extraction, 100 mg of pulverized leaf material of each sample preserved in NaCl-CTAB was used.

### DNA extraction and quantification

DNA was extracted using the DNeasy Plant Mini Kit (QIAGEN, Hilden, Germany). Genomic DNA quality and quantity were checked by 1% (w/v) agarose gel electrophoresis and using a NanoDrop spectrophotometer. Lastly, the DNA concentration was measured with the Qubit^®^ dsDNA BR assay kit (Q) and Quant-iT™ PicoGreen™ (Life Sciences) dsDNA assay.

### Double-digest restriction-site-associated DNA library preparation and llumina sequencing

The ddRAD procedure used in this study was modified from (Peterson et al., 2012). We calculated the number of reads required for 20X coverage of restriction fragments in the 150–500bp size range across 10 multiplexed individuals using multiple enzyme pairs, assuming a GC content of 0.44, to ensure that restriction fragments could feasibly be sequenced with enough coverage on an Illumina MiSeq platform. Five hundred ng of each DNA sample was double digested using *EcoR1* and *MseI* restriction endonucleases, and unique P1 barcode adapters were ligated to the digested fragments from each sample while a common P2 barcode adapter was ligated to fragments from all samples (For information about adapters and primers, see **Supplementary Tables 2**-**4**). Samples containing unique P1 barcodes were pooled, and the Sage Science Blue Pippin system (www.sagescience.com) was used to select fragments of about 500bp. Size-selected libraries were bound to Dynabeads^®^ M-270 Streptavidin magnetic beads (Invitrogen) to eliminate fragments without the P2 adapter, and the libraries amplified by PCR using Phusion™ Polymerase kit (Invitrogen) and index-marked primers for further tagging of the samples (**Supplementary Table 4**). The libraries were analyzed using an Agilent 2100 BioAnalyser and diluted to a concentration of 35nM for sequencing using the V2 sequencing kit on the MiSeq platform (Illumina). The paired end sequencing of 200 base pair was performed using Illumina MiSeq at the Norwegian University of Life Sciences, Ås, Norway.

### Sequence data analysis and SNP calling

The ddRAD sequence data obtained was quality checked using the FastQC program (v0.11.9) (Andrews, 2010). High quality reads were retained after trimming the bad quality reads using the Trimmomatic program (v0.39) (Bolger et al., 2014) using the options *SLIDINGWINDOW:4:25* and *MINLEN:35*. The SNP calling was performed using STACKS (v2.4) (Rochette et al., 2019). The *denovo_map.pl* script from the STACKS program was used to assemble and analyze loci *de novo*, without a reference genome. The following parameters were used for the SNP calling: *-m 3* (minimum depth of coverage required to create a stack, a set of short read sequences that align to a given locus set to 3); *-M 4* (number of mismatches allowed between sample loci when building the catalog); *-n 4* (number of mismatches allowed between loci when analyzing a population to call a heterozygote or homozygote); *-X “populations: -p 3 -r 0.80”* (*-p 3* requires at least 3 populations to have a polymorphic locus for it to be processed, and *-r 0.80* sets the minimum percentage of individuals in a population required to process a locus for that population to 80%. The obtained SNPs were further quality filtered using VCFTools (version 4.1) (Danecek et al., 2011) according to the following criteria: (1) variants should be bi-allelic SNPs, (2) SNPs having more than 20% missing information were excluded, (3) genotypes having more than 20% missing information were excluded, and (4) markers with minor allele frequency (MAF; MAF > 0.05) were retained.

### Genetic diversity, population structure and phylogenetic analyses

Genetic variation among and within populations/regions, observed (H_o_) and expected (H_e_) heterozygosity, pairwise fixation indices (F_ST_) (Weir and Cockerham, 1984) for the three subpopulations, and molecular variance (AMOVA) was estimated using the *adegenet* R package (v2.1.10) (Jombart and Ahmed, 2011). To examine the relationship among Entada, and cultivated and wild enset (365 genotypes altogether), principal component analyses (PCA) were performed using TASSEL v5.2 (Bradbury et al., 2007) and maximum-likelihood (ML) phylogenetic tree analyses performed using PhyML 3.0 (Guindon et al., 2010). Only SNP loci were used for the ML analysis. The SMS (Smart Model Selection) software tool in PhyML (Lefort et al., 2017) was used to determine the evolutionary model with the best fit using the Bayesian Information Criterion (BIC). The trees were prepared and visualized using the iTOL v4 online tool (Letunic and Bork, 2019). Population structure was estimated using fastSTRUCTURE (Raj et al., 2014) as described in Haile et al. (2023).

### F_ST_ outlier analysis to detect candidate genes for sucker development

Sucker formation is expected to be a strongly selected trait, and therefore SNP variation associated to it is likely to be flagged as an F_ST_ outlier. Therefore, to identify genes that might be involved in sucker formation, we performed genome scans using F_ST_ outlier analysis. We used the hierarchical method (Excoffier et al., 2009), a modified approach of Beaumont and Nichols (1996) implemented in the Arlequin software package version 3.5.1.3 (Excoffier and Lischer, 2010) to detect loci under directional selection. We conducted hierarchical island model simulations on two populations, cultivated enset (226 genotypes) and Entada (117 genotypes) with 50,000 simulations to generate the joint distribution of F_ST_ versus heterozygosity using 2,823 polymorphic SNP markers (see Results section). We also did this simulation comparing the 226 cultivated enset genotypes and the 12 genotypes that looks morphologically as Entada. Loci that fall outside the 99% confidence intervals of the distribution were identified as outliers being putatively under selection. The putative function of genes with outlier SNPs was identified using the Gene Ontology (GO) annotation using Blast2GO software tool version 3.0 (Conesa et al., 2005). The identification of putative genes using outlier SNP data in Blast2GO without a benchmark or reference annotated genome was based on an integrative approach that combined outlier SNP identification, followed by retrieving the flanking sequences of the outlier SNPs. The flanking sequences of the SNPs were queried against a local database created using the NCBI non-redundant (nr) database specifically filtered to include only sequences from the Viridiplantae clade (downloaded on 19.08.2022), using blastn to identify the predicted functions, and functional annotation analysis. The criteria for selecting putative genes involved: identifying SNPs with significant deviation in frequency, mapping these SNPs to genomic regions or predicted genes, annotating these genes based on sequence similarity to known gene databases, and selecting genes based on their potential biological significance and statistical robustness. A False Discovery Rate (FDR) of 0.05 was applied to account for multiple testing and control the proportion of false positives.

## Results

### SNP markers developed for Entada

The sequencing of all Entada ddRADs libraries produced a total of 2.133 billion raw reads, corresponding to an average of 5.86 M read pairs per sample. About 1.6 M low-quality reads and adapter sequences were filtered out resulting in about 4.26 M high-quality reads per sample used for identifying SNPs. A total of 160,000 SNPs were detected before filtering. After filtering, 1,617 high quality SNP markers remained.

### Phylogenetic and population structure analyses

**Figure 3:**
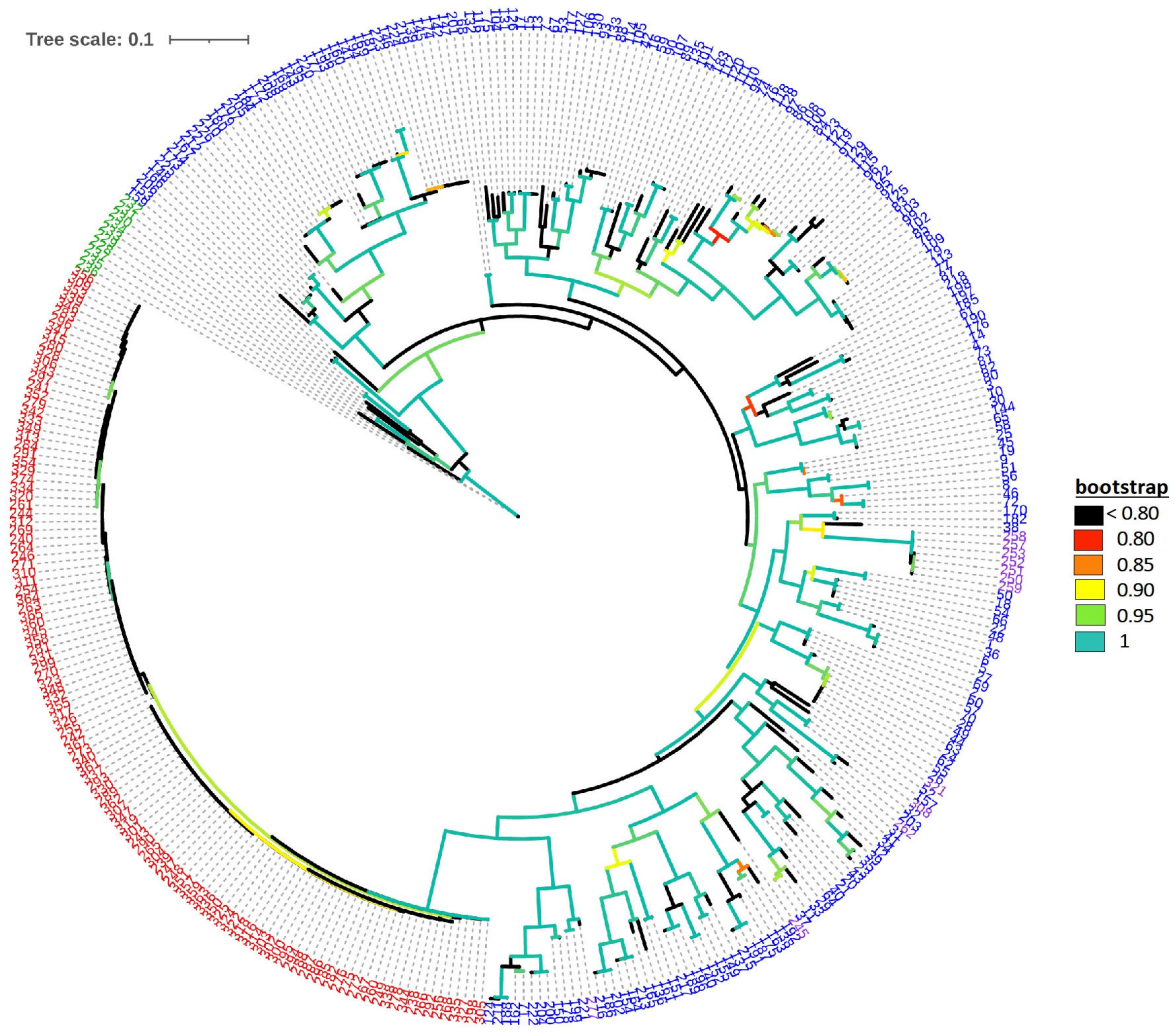
Maximum likelihood phylogenetic tree with branch lengths. Genotype numbers (ID) are colored according to their propagation method and cultivation status, i.e., blue: 226 cultivated enset genotypes (ID 1-227, ID 98 missing); green: 10 wild enset genotypes (ID 228-237), red: 117 Entada genotypes (ID 238-367, except IDs 245, 250, 251, 252, 253, 257, 258, 259, 262, 277, 308, 321 which are determined to be cultivated ensets with suckers, ID 366 missing), and purple: the 12 cultivated enset with suckers.

**Figure 4:**
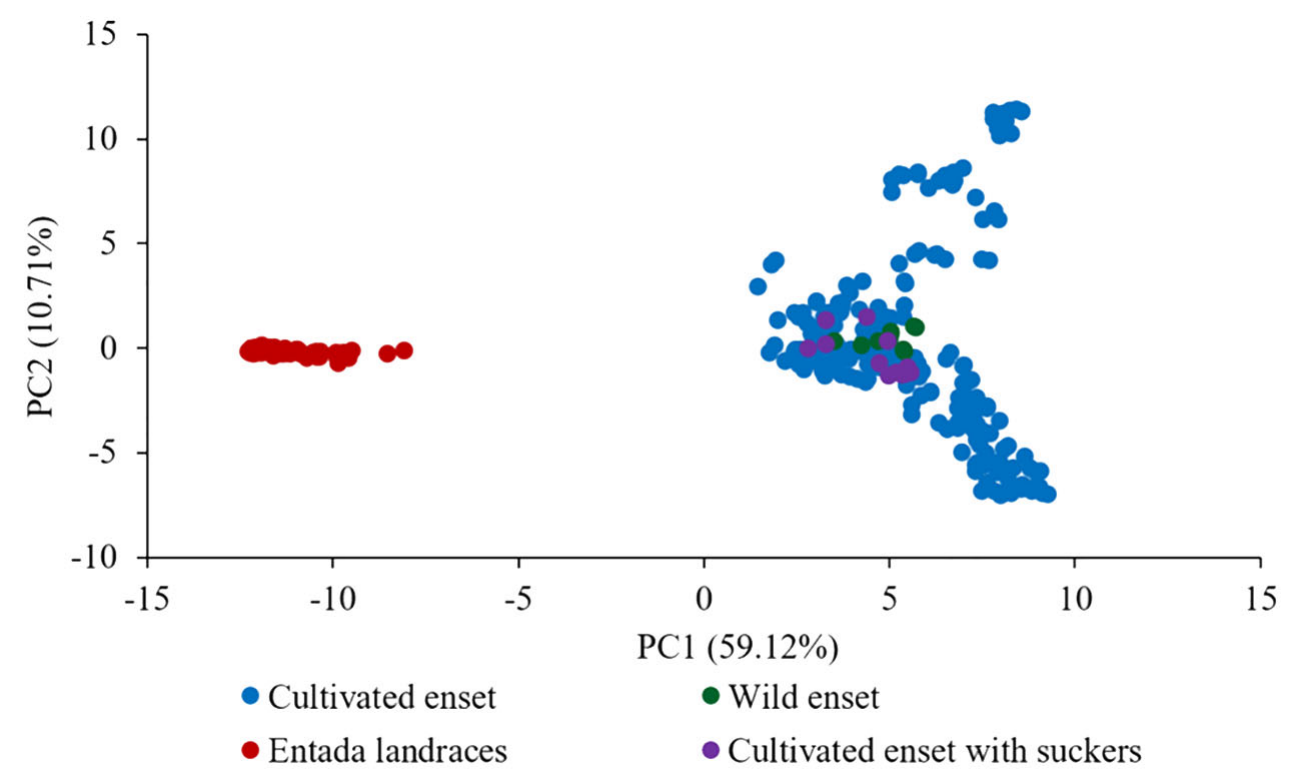
Principal component analysis (PCA) of 365 genotypes of different enset types (226 cultivated, 10 wild, 12 cultivated enset with suckers and 117 Entada). The percentages along the axes denote the variances explained by the two PCs.

**Figure 5:**
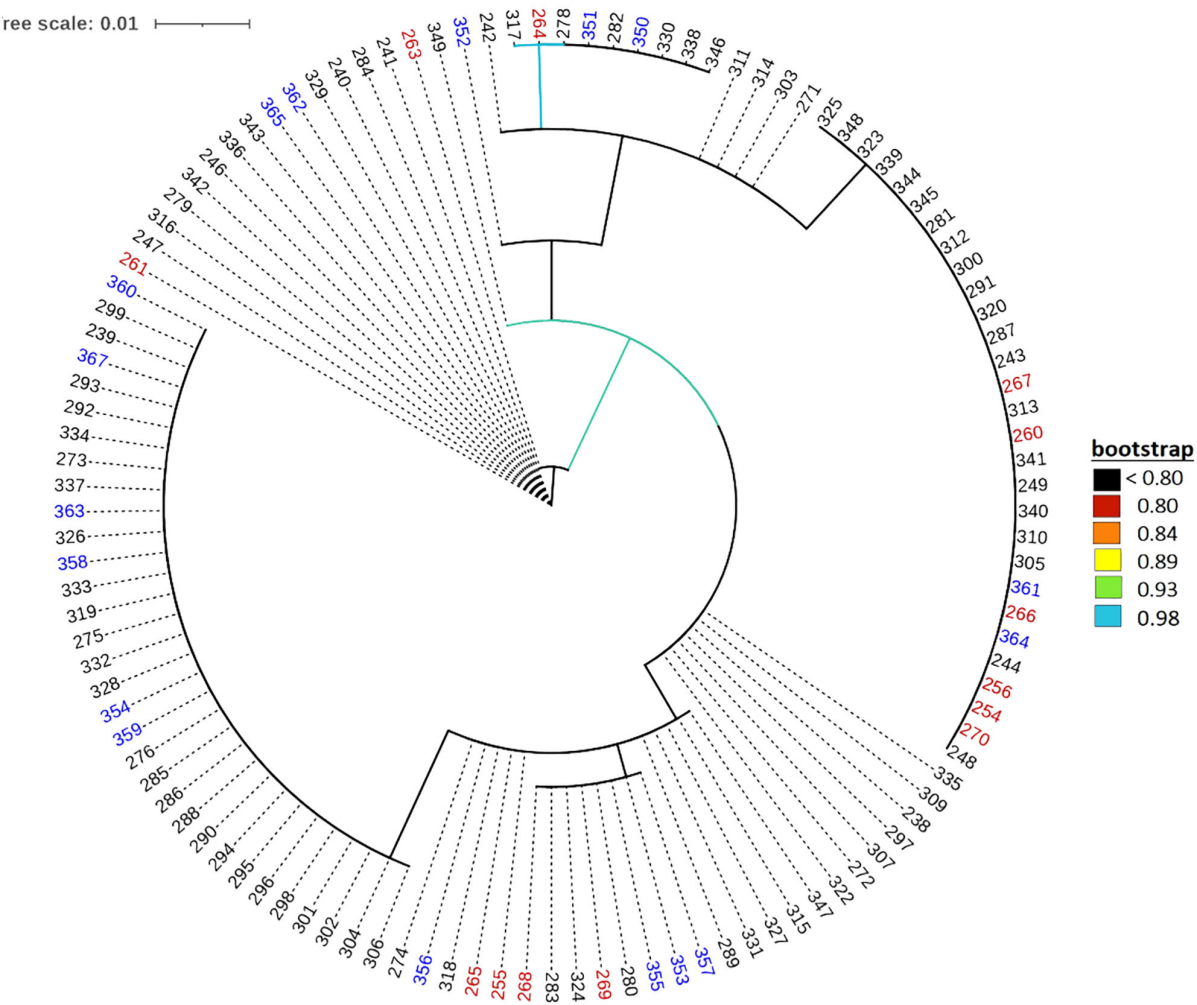
Maximum likelihood phylogenetic tree of the 117 Entada genotypes. The colors of the genotypes indicate their geographical origin, i.e., South Ari: black; Sidama: red; North Ari: blue.

**Figure 6:**
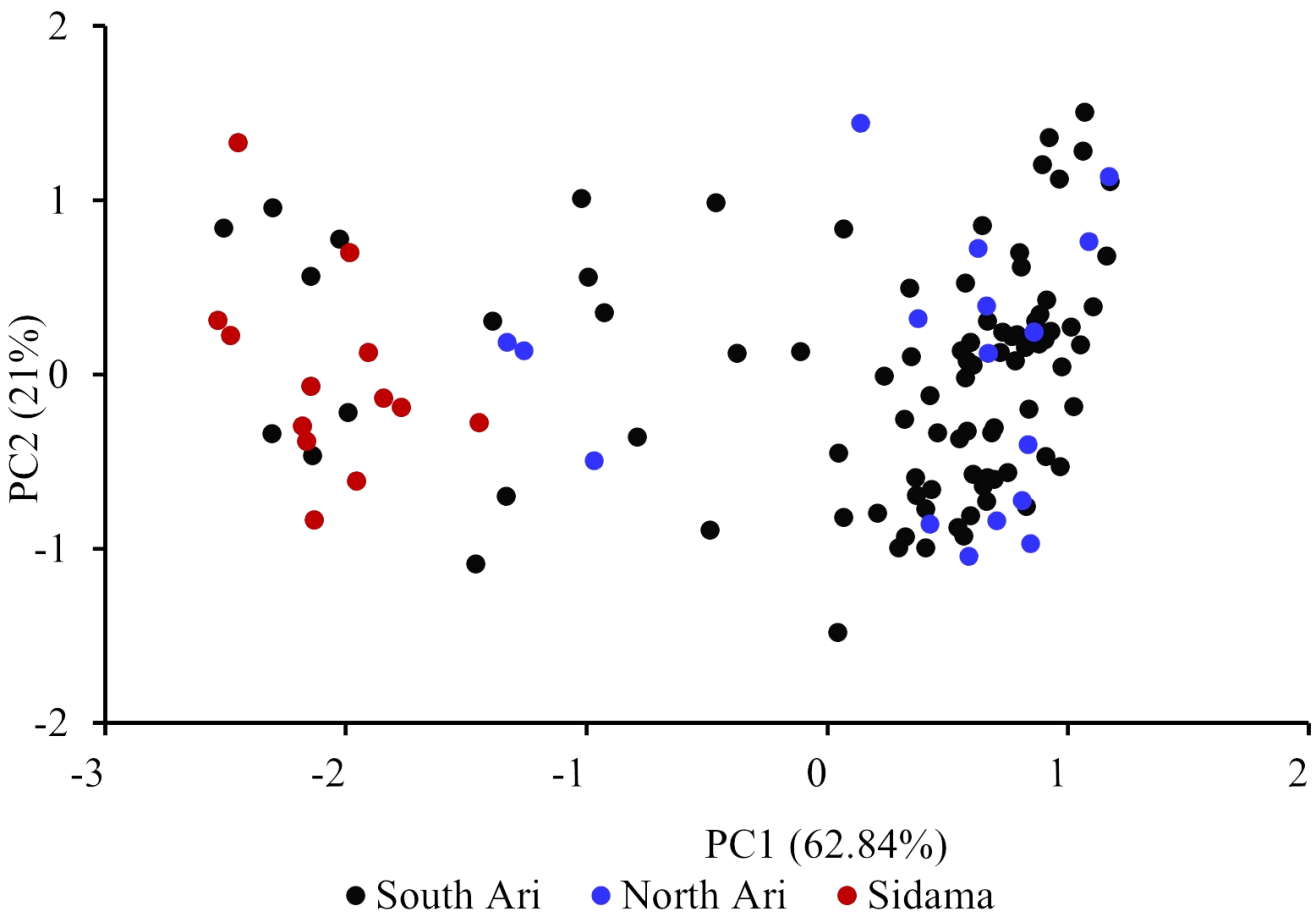
Principal component analysis (PCA) of the 117 Entada genotypes. The percentages along the axes denote the variances explained by the two PCs.

The default HKY85 substitution model was selected based on the (Smart Model Selection) software tool in PhyML (Lefort et al., 2017). The joint phylogenetic analysis involving all enset genotypes grouped the cultivated enset, wild enset and Entada genotypes into three distinct clades (**Figure 3** **).** However, 12 genotypes collected as Entada genotypes (8 from Sidama and 4 from North Ari) cluster with cultivated enset. These genotypes produce suckers and resemble Entada phenotypically. The PCA analysis confirms that Entada is completely different from other ensets, while cultivated enset, cultivated enset with suckers and wild enset group together (**Figure 4**) with PC1 and PC2 accounting for 59.12, and 10.71 of the variation, respectively. Phylogenetic analysis of the Entada genotypes showed several clusters/branches (**Figure 5**). One genotype from Sidama is clearly separated from other clusters. Ten genotypes from South Ari and two from North Ari clustered together. Several genotypes from South Ari, North Ari and Sidama cluster together. The phylogenetic analysis did not show clear differentiation between the regions. The fasSTRUCTURE analysis found no population structure, the Entada genotypes formed a single group, while two separate groups, i.e., Entada and cultivated enset with suckers, were identified when the structure analysis was performed on the complete Entada collection (129 genotypes, **Supplementary Figure 1**). However, the PCA analysis showed that Entada from Sidama and North Ari was completely separated, while Entada from Sidama partly overlapped with Entada from South Ari (**Figure 6**). PC1 and PC2 explain 62.84 and 21% of the variation, respectively.

### Genetic differentiation of enset and genetic diversity of Entada

AMOVA analysis was performed among and within the three enset types cultivated enset, wild enset and Entada using the 2,823 common SNP markers (**Table 1**). The molecular variation was distributed with 51.3% among enset types and 48.7% within. Observed heterozygosity (H_o_) was highest among the Sidama genotypes (0.79), followed by South Ari (0.73) and lowest in North Ari (0.67), while the corresponding expected heterozygosities (H_e_) was 0.52, 0.50 and 0.47, respectively (**Table 2**). Average pairwise F_ST_ show that the differentiation (allele frequency differences) was substantial between South Ari and Sidama (0.4), relatively large (0.2) between North Ari and Sidama, while it was low between the two Ari regions (0.1) (**Table 3**).

**Table 1:** Analysis of Molecular Variance (AMOVA) among and within enset types (cultivated, wild and Entada) based on 2,823 common SNP markers with 5,646 alleles in 365 genotypes (238 cultivated, 10 wild and 117 Entada genotypes).

Source	df	Sum of squares	Mean squares	Variance component	Variance %
Among enset types	2	15,073.47	7,536.73	86.66	51.3
Within enset types	362	29,783.58	82.28	82.28	48.7
Total	364	44,857.05	123.23	168.94	

**Table 2:** Estimates of observed and expected heterozygosities of Entada genotypes from three regions in Ethiopia.

Population	No. of genotypes	Observed (H_o_)	Expected (H_e_)
South Ari	87	0.73	0.50
North Ari	17	0.67	0.47
Sidama	13	0.79	0.52

**Table 3:** Average pairwise population differentiation (F_ST_).

Population	South Ari	North Ari
North Ari	0.1	
Sidama	0.4	0.2

### Loci under selection

**Figure 7:**
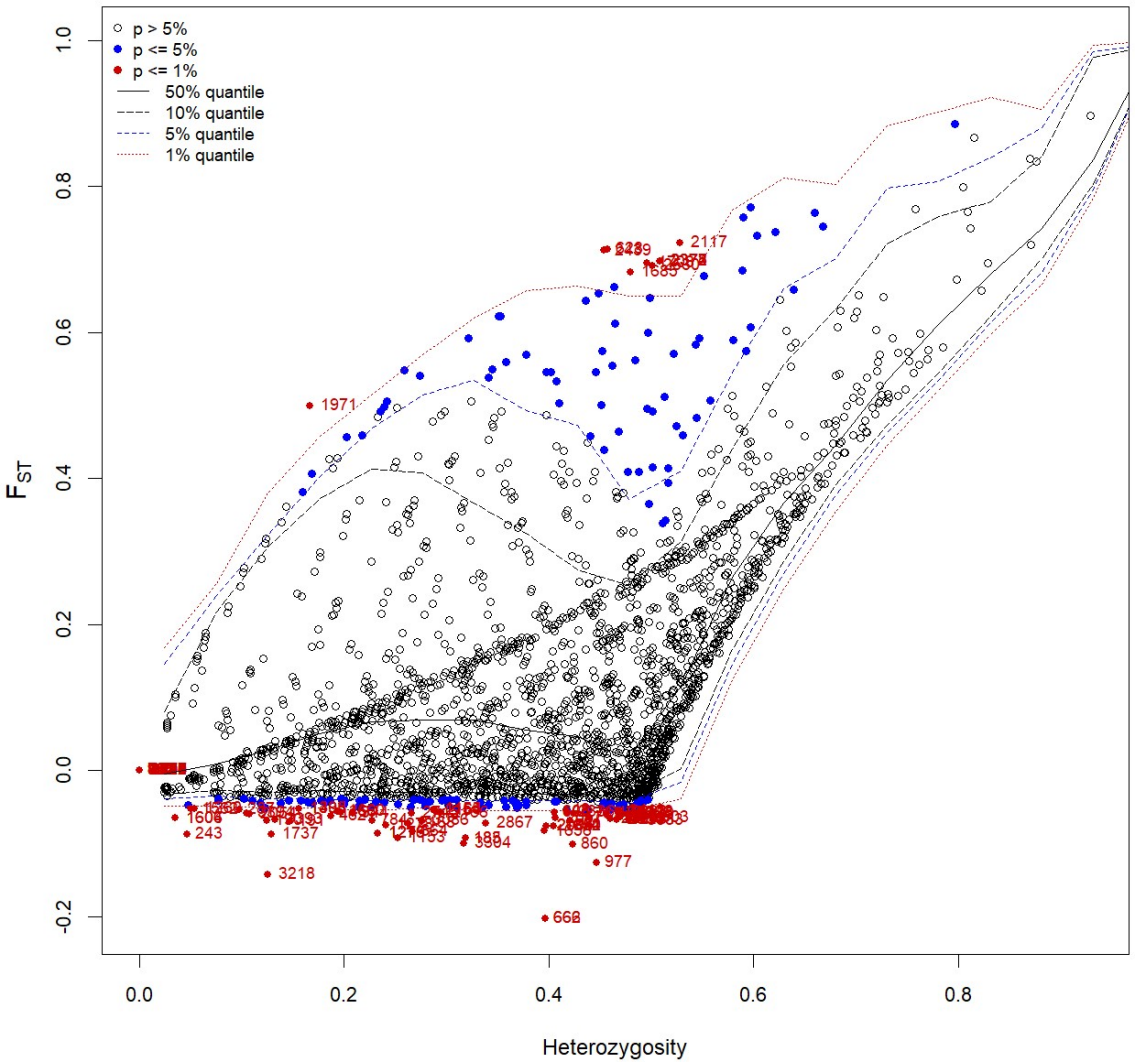
Candidate loci under selection were identified using FST based outlier approach (Hierarchical structure model using Arlequin 3.5). FST: locus–specific genetic divergence among the populations; Heterozygosity: measure of heterozygosity per locus. Loci significant at the 1% level are indicated by red dots.

Based on the F_ST_ outlier analysis of cultivated enset and Entada, we identified eight candidate loci under positive selection and 72 loci under balancing selection based on F_ST_ values that displayed differentiation higher than the 99% limit of the confidence interval (**Figure 7**; **Table 4**). Among the eight loci, four have putative gene functions, i.e., *Lateral suppressor protein, Auxin response factor 2A, Cytokinin dehydrogenase*, and *Scarecrow-like protein 18* (**Table 4** **).** The analysis comparing the 12 cultivated ensets morphologically similar to Entada with the other cultivated ensets did not identify any significant outliers, likely due to the limited number of genotypes being compared. This result underscores the need for a broader sampling to detect potential genetic differences associated with sucker formation.

**Table 4:** Candidate genes under directional selection and potentially involved in sucker formation, detected using F_ST_ outlier analysis.

SNP ID	Gene name	Gene function	Reference
E-1971	Lateral suppressor protein	Role in secondary shoot formation	https://www.uniprot.org/uniprot/B5M4A5
E-2117	Auxin response factor 2A	Regulates vegetative growth, lateral root formation and flower organ senescence	https://www.uniprot.org/uniprot/Q2LAJ3
E-1685	Cytokinin dehydrogenase	Play a key role in plant growth and development including maintenance of root and shoot meristems	https://www.uniprot.org/uniprot/A0A1S4ARV5
E-2580	Scarecrow-like protein 18	Transcription factor required for axillary (lateral) shoot meristem formation during vegetative development	https://www.uniprot.org/uniprot/Q9ZWC5

## Discussion

### Genetic relationships and diversity among enset types

Previously we developed 3,505 high quality SNP markers which were polymorphic in both cultivated and wild enset (Haile et al., 2023). Of these, 2,823 SNP markers were polymorphic across all three enset types (226 cultivated, 10 wild and 129 Entada genotypes) and used to perform a phylogenetic analysis.

The phylogenetic analysis detected a clear distinction between cultivated enset, Entada and wild enset (**Figure 3**). This was also evident in the AMOVA analysis with 51.3% of the molecular variation present among the three enset types (**Table 1**).Usually, most of the molecular variation would exist within populations and individuals, especially with clonal species. In this case the very distinctive differentiation between the three enset types contributes to a larger than normal variance among enset types (51.3%), but there is still a lot of molecular variation among genotypes within the three enset types, mostly within cultivated and wild enset. The genetic differentiation of cultivated and wild enset, has to a large degree been explained by cultivation status and reproduction methods (Birmeta et al., 2004; Gerura et al., 2019; Olango et al., 2015). Wild enset regenerates from botanical seeds and hence lack spontaneous suckers, while cultivated enset and Entada are propagated vegetatively by suckers. However, formation of suckers is different; suckers are induced in cultivated enset, while in Entada suckers are formed spontaneously (Bekele and Shigeta, 2011; Olango et al., 2015). Our results demonstrate that there are exceptions to this general rule. We found that twelve of the Entada genotypes clustered together with cultivated enset (**Figure 3**). Observations in the field confirm that these Entada genotypes phenotypically look like cultivated enset with natural suckers. Therefore, it is important to combine phenotypic and molecular characterization of diverse ensets collected for conservation and utilization in gene banks. In addition to the differences in reproduction and propagation, various factors like long-term evolutionary history, genetic drift, gene flow and selection (Godoy et al., 2018; Schaal et al., 1998) have contributed to shaping the present genetic structure of enset types.

### Genetic structure of Entada

Balloux et al. (2003) simulated the effect of clonal or partial clonal reproduction on the population genetics of neutral markers in diploid organisms. They found that high rates of clonal reproduction increase heterozygosity, maintain higher genetic diversity at each single locus while the genotypic diversity is reduced. Population size also increases towards extreme since the polymorphism is protected within individuals due to fixed heterozygosity. Our observations are very much in line with these theoretical predictions. We found that observed heterozygosity (H_o_) was high (weighted average 0.73) in the Entada genotypes, and the expected heterozygosity (H_e_) intermediate (weighted average 0.50). Similar estimates of heterozygosity were found in a study of genetic diversity of landraces of the related species plantain (*Musa sapientum* L.) from Côte d’Ivoire using SSR marker*s* (Cyrille et al., 2019). However, extremely short branch lengths as evident from the phylogenetic analysis (**Figure 3**), indicate very little molecular evolution among the Entada genotypes. Overall, our results strongly indicate that the Entada genotypes we have studied originate from one or a few clonal lineages that have been propagated and spread among farmers as clones.

Absence of sexual reproduction will promote divergence between alleles within loci, as the two copies will accumulate different mutations over time (Judson and Normark, 1996; Rousset, 2002). This has been partly confirmed in recent study of the domestication of enset landraces using tGBS derived SNP markers by White et al. (2023). They found reduced heterozygosity in cultivated enset indicating a domestication bottleneck, but an accumulation of putatively deleterious mutations at low frequency present as heterozygotes. The current cultivated enset originates most probably from historic sexual recombination and subsequent long-term clonal propagation of favorable genotypes (White et al., 2023). While cultivated enset consists of several domesticated lineages, our study of Entada indicates that the genotypes originate from a single or very few original mutant lineages within enset. Since sexual reproduction is absent, the strict and easy clonal propagation by natural suckers has preserved the genetic structure of Entada with high levels of heterozygosity within and very little between genotypes. White et al. (2023) reported a heterozygosity of 0.067 for cultivated enset which is very low compared to our estimates of about 0.73 for Entada. However, these estimates cannot be compared since the estimate for cultivated enset is the proportion of heterozygous sites per individual (nucleotide diversity) while our estimate is a measure of heterozygosity per polymorphic SNP site.

Observed heterozygosity (H_o_) was highest among the Sidama genotypes (0.79), and the average pairwise F_ST_ was also much higher between Sidama and South Ari (0.4) compared to the other pairwise comparisons (**Table 3**). The Sidama genotypes we studied were sampled from the germplasm collection at the Hawassa University field station. This collection contains Entada of different origins, some from South Ari and others from Addis Ababa. The Addis Ababa genotypes are certainly not from the city but has been brought there from elsewhere, probably from the Ari regions. The 13 Sidama genotypes we used overlap with some of the South Ari genotypes in the PCA plot (**Figure 6**), confirming their origin. However, the bulk of the South Ari genotypes group with North Ari genotypes completely separated from the Sidama/South Ari group along the PC1 axis, and this explains the higher F_ST_ estimate between Sidama and South Ari. The structure analysis found no structure within the Entada genotypes, only between the Entada and the cultivated ensets with suckers, corroborating the results of the phylogenetic and PCA analyses.

### Identification of potential candidate genes for sucker development

F_ST_ outlier approaches were applied in studies in many crops, i.e., tomato (Sim et al., 2011), European beech (Cuervo-Alarcon et al., 2018), soybean (Li et al., 2014), banana (Sardos et al., 2022), and common bean (Papa et al., 2007) for identifying adaptive differentiations. Markers detected in these crops are mapped to the genomic regions with known QTL/genes related to domestication. These loci may be directly under selection. Putative functions of the candidate SNP loci detected in the present study revealed that at least four genes with known function are involved in axillary shoot formation. Genes involved in axillary meristem development were studied in various plant species. Mutations in the *Lateral suppressor* genes in *Arabidopsis* (LAS) (Greb et al., 2003) and in tomato (*Ls*) (Schumacher et al., 1999) inhibits axillary shoot formation during the vegetative phase. Further, it is well known that the phytohormones, auxin and cytokinin interact to regulate many plants growth and developmental processes (Schaller et al., 2015). Functional characterization of some auxin response factors based on the phenotypes of the loss-of-function and gain-of-function mutants showed abnormal abscission of the floral organs (Ellis et al., 2005) and impaired hypocotyl elongation and auxin homeostasis (Goetz et al., 2006), while the tomato *SlARF3* was found to participate in the formation of trichomes and epidermal cells (Zhang et al., 2015). The cytokinins, which are positive regulators of shoot growth and negative regulators of root growth (Werner et al., 2003), are implicated in the control of the shoot architecture (Han et al., 2014). Thus, candidate genes detected in this study are involved in axillary growth and they might have important influences on the natural and induced sucker formation in Entada and enset populations. The 72 loci detected to be under balancing selection are not involved in the genetic regulation of sucker formation. Balancing selection will maintain genetic diversity for various reasons that may not be directly related to the trait we are investigating. Therefore, while these loci are of interest, they do not appear to significantly contribute to the specific phenotypic differences between ensets with and without suckers.

The F_ST_ outlier analysis comparing cultivated enset morphologically similar to Entada with the other cultivated ensets, would potentially be most powerful in detecting genes involved in sucker formation. However, this analysis yielded no significant outliers, which we believe is due to the small and unbalanced sample sizes (12 ensets with suckers vs. 226 normal ensets). While our findings identify interesting candidate genes for sucker formation, it is highly likely that a larger sample of cultivated enset with suckers would make it possible to understand the genetic basis of sucker formation in enset, which constitutes the main difference between Entada and the cultivated enset.

## Conclusion

To the best of our knowledge, this study is the first application of SNP markers to study molecular diversity in genotypes of Entada in Ethiopia. The results clearly demonstrate that there is very little clonal diversity among Entada genotypes from the three regions in Ethiopia. It confirms that Entada is naturally propagated by spontaneous suckers. Limited genetic diversity among Entada genotypes and the relatively low genetic structure in the main growing regions in South and North Ari provide an important basis for developing strategies for the conservation of this enset landrace in the active germplasm bank in Ethiopia. Furthermore, we identified genes that might be involved in axillary shoot growth crucial for sucker formation. Knowledge about the genetic regulation of sucker formation is important for characterization of germplasm and for developing breeding strategies.

## Data Availability

The datasets presented in this study can be found in online repositories. The names of the repository/repositories and accession number(s) can be found below: https://datadryad.org/stash, doi: 10.5061/dryad.4qrfj6qhs.

## Supplementary material

Supplementary Figure 1 Population structure of the 117 Entada genotypes and the 12 cultivated enset with suckers. **(A)** The model complexity that maximizes likelihood is 2. The highest peak shows the best K = 2. **(B)** Population structure based on fastSTRUCTURE output resulting in K = 2 being the most likely number of genetic clusters. Entada genotypes = red; cultivated enset with suckers = aqua.

## References

[B1] Andrews S. (2010). FastQC A Quality Control tool for High Throughput Sequence Data: Babraham Bioinformatics. Available online at: https://www.bioinformatics.babraham.ac.uk/projects/fastqc/.

[B2] Baird, N. A. Etter P. D. Atwood T. S. Currey M. C. Shiver A. L. Lewis Z. A.. (2008). Rapid SNP Discovery and Genetic Mapping Using Sequenced RAD Markers. PLoS ONE, 3(10), e3376. doi: 10.1371/journal.pone.0003376 PMC2557064 18852878

[B3] Balloux F. Lehmann L. de Meeûs T. (2003). The population genetics of clonal and partially clonal diploids. Genetics 164, 1635–1644. doi: 10.1093/genetics/164.4.1635 12930767 PMC1462666

[B4] Beaumont M. A. Nichols R. A. (1996). Evaluating loci for use in the genetic analysis of population structure. Proc. R. Soc. London. Ser. B: Biol. Sci. 263, 1619–1626. doi: 10.1098/rspb.1996.0237

[B5] Bekele E. Shigeta M. (2011). Phylogenetic relationships between *Ensete* and *Musa* species as revealed by the trnT trnF region of cpDNA. Genet. Resour. Crop Evol. 58, 259–269. doi: 10.1007/s10722-010-9568-2

[B6] Birmeta G. Nybom H. Bekele E. (2004). Distinction between wild and cultivated enset ( *Ensete ventricosum* ) gene pools in Ethiopia using RAPD markers. Hereditas 140, 139–148. doi: 10.1111/j.1601-5223.2004.01792.x 15061792

[B7] Bolger A. M. Lohse M. Usadel B. (2014). Trimmomatic: a flexible trimmer for Illumina sequence data. Bioinformatics 30, 2114–2120. doi: 10.1093/bioinformatics/btu170 24695404 PMC4103590

[B8] Bradbury P. J. Zhang Z. Kroon D. E. Casstevens T. M. Ramdoss Y. Buckler E. S. (2007). TASSEL: software for association mapping of complex traits in diverse samples. Bioinformatics 23, 2633–2635. doi: 10.1093/bioinformatics/btm308 17586829

[B9] Brandt S. Spring A. Hiebsch C. McCabe J. Tabogie E. Diro M.. (1997). The “Tree Against Hunger”: Enset-based agricultural systems in Ethiopia. Vol. 56 (Washington, DC, USA: American Association for the Advancement of Science).

[B10] Brown A. (1978). Isozymes, plant population genetic structure and genetic conservation. Theor. Appl. Genet. 52, 145–157. doi: 10.1007/BF00282571 24317500

[B11] Campbe B. (1987). The use of wild fruits in Zimbabwe. Econ Bo 41, 375–385. doi: 10.1007/BF02859054

[B12] Conesa A. Götz S. García-Gómez J. M. Terol J. Talón M. Robles M. (2005). Blast2GO: a universal tool for annotation, visualization and analysis in functional genomics research. Bioinformatics 21, 3674–3676. doi: 10.1093/bioinformatics/bti610 16081474

[B13] Cyrille K. K. G. Désiré P. Sylvère S. R. Fulgence T. D. E. (2019). Genetic diversity and structure of plantain (*Musa sapientum* L.) landraces from Côte d’Ivoire using SSR markers. Eur. J. Biotechnol. Bioscience 7, 36–43.

[B14] Cuervo-Alarcon L. Arend M. Müller M. Sperisen C. Finkeldey R. Krutovsky K. V. (2018). Genetic variation and signatures of natural selection in populations of European beech (*Fagus sylvatica* L.) along precipitation gradients. Tree Genetics & Genomes, 14(6). doi: 10.1007/s11295-018-1297-2

[B15] Danecek P. Auton A. Abecasis G. Albers C. A. Banks E. DePristo M. A.. (2011). The variant call format and VCFtools. Bioinformatics 27, 2156–2158. doi: 10.1093/bioinformatics/btr330 21653522 PMC3137218

[B16] Davik J. Sargent D. J. Brurberg M. B. Lien S. Kent M. Alsheikh M.. (2015). A ddRAD Based Linkage Map of the Cultivated Strawberry, Fragaria x ananassa. PLoS ONE, 10(9), e0137746. doi: 10.1371/journal.pone.0137746 PMC4580419 26398886

[B17] Ellis C. M. Nagpal P. Young J. C. Hagen G. Guilfoyle T. J. Reed J. W. (2005). AUXIN RESPONSE FACTOR1 and AUXIN RESPONSE FACTOR2 regulate senescence and floral organ abscission in *Arabidopsis thaliana* . Development, 132(20), 4563–4574. doi: 10.1242/dev.02012 16176952

[B18] Esposito S. Cardi T. Campanelli G. Sestili S. Díez M. J. Soler S.. (2020). ddRAD sequencing-based genotyping for population structure analysis in cultivated tomato provides new insights into the genomic diversity of Mediterranean ‘da serbo’ type long shelf-life germplasm. Horticulture Research, 7(1), 134. doi: 10.1038/s41438-020-00353-6 PMC7459340 32922806

[B19] Excoffier L. Hofer T. Foll M. (2009). Detecting loci under selection in a hierarchically structured population. Heredity 103, 285–298. doi: 10.1038/hdy.2009.74 19623208

[B20] Excoffier L. Lischer H. E. (2010). Arlequin suite ver 3.5: a new series of programs to perform population genetics analyses under Linux and Windows. Mol. Ecol. Resour. 10, 564–567. doi: 10.1111/j.1755-0998.2010.02847.x 21565059

[B21] Gerura F. N. Meressa B. H. Martina K. Tesfaye A. Olango T. M. Nasser Y. (2019). Genetic diversity and population structure of enset (*Ensete ventricosum* Welw Cheesman) landraces of Gurage zone, Ethiopia. Genet. Resour. Crop Evol. 66, 1813–1824. doi: 10.1007/s10722-019-00825-2

[B22] Godoy F. M. d. R. Lenzi M. Ferreira B. H. D. S. Silva L. V. D. Zanella C. M.. (2018). High genetic diversity and moderate genetic structure in the self-incompatible, clonal *Bromelia hieronymi*(*Bromeliaceae*). Botanical J. Linn. Soc. 187, 672–688. doi: 10.1093/botlinnean/boy037

[B23] Goetz M. Vivian-Smith A. Johnson S. D. Koltunow A. M. (2006). AUXIN RESPONSE FACTOR8 is a negative regulator of fruit initiation in *Arabidopsis* . Plant Cell 18, 1873–1886. doi: 10.1105/tpc.105.037192 16829592 PMC1533983

[B24] Greb T. Clarenz O. Schäfer E. Müller D. Herrero R. Schmitz G.. (2003). Molecular analysis of the LATERAL SUPPRESSOR gene in *Arabidopsis* reveals a conserved control mechanism for axillary meristem formation. Genes Dev. 17, 1175–1187. doi: 10.1101/gad.260703 12730136 PMC196050

[B25] Guindon S. Dufayard J.-F. Lefort V. Anisimova M. Hordijk W. Gascuel O. (2010). New algorithms and methods to estimate maximum-likelihood phylogenies: assessing the performance of PhyML 3.0. Systematic Biol. 59, 307–321. doi: 10.1093/sysbio/syq010 20525638

[B26] Haile A. T. Kovi M. R. Johnsen S. S. Tesfaye B. Hvoslef-Eide T. Rognli O. A. (2023). Genetic diversity, population structure and selection signatures in Enset (*Ensete ventricosum*, (Welw.) Cheesman), an underutilized and key food security crop in Ethiopia. Genet. Resour. Crop Evol. 71(3), 1159–1176. doi: 10.1007/s10722-023-01683-9

[B27] Han Y. Yang H. Jiao Y. (2014). Regulation of inflorescence architecture by cytokinins. Front. Plant Sci. 5. doi: 10.3389/fpls.2014.00669 PMC4241816 25505480

[B28] Hawkes J. G. (1983). The diversity of crop plants (Harvard University Press). doi: 10.4159/harvard.9780674183551

[B29] Hinze L. L. Hulse-Kemp A. M. Wilson I. W. Zhu Q.-H. Llewellyn D. J. Taylor J. M.. (2017). Diversity analysis of cotton (*Gossypium hirsutum* L.) germplasm using the CottonSNP63K Array. BMC Plant Biol. 17, 37. doi: 10.1186/s12870-017-0981-y 28158969 PMC5291959

[B30] Jin Y. Zhao W. Nie S. Liu S.-S. El-Kassaby Y. A. Wang X.-R.. (2019). Genome-Wide Variant Identification and High-Density Genetic Map Construction Using RADseq for Platycladus orientalis (Cupressaceae). G3 Genes|Genomes|Genetics, 9(11), 3663–3672. doi: 10.1534/g3.119.400684 PMC6829139 31506321

[B31] Jombart T. Ahmed I. (2011). adegenet 1.3-1: new tools for the analysis of genome-wide SNP data. Bioinformatics 27, 3070–3071. doi: 10.1093/bioinformatics/btr521 21926124 PMC3198581

[B32] Judson O. P. Normark B. B. (1996). Ancient asexual scandals. Trends Ecol. Evol. 11, 41–46. doi: 10.1016/0169-5347(96)81040-8 21237759

[B33] Konar A. Choudhury O. Bullis R. Fiedler L. Kruser J. M. Stephens M. T.. (2017). High-quality genetic mapping with ddRADseq in the non-model tree Quercus rubra. BMC Genomics, 18(1), 417. doi: 10.1186/s12864-017-3765-8 PMC5450186 28558688

[B34] Kölliker R. Herrmann D. Boller B. Widmer F. (2003). Swiss Mattenklee landraces, a distinct and diverse genetic resource of red clover (*Trifolium pratense* L.). Theor. Appl. Genet. 107, 306–315. doi: 10.1007/s00122-003-1248-6 12845445

[B35] Lefort V. Longueville J.-E. Gascuel O. (2017). SMS: smart model selection in phyML. Mol. Biol. Evol. 34, 2422–2424. doi: 10.1093/molbev/msx149 28472384 PMC5850602

[B36] Letunic I. Bork P. (2019). Interactive Tree Of Life (iTOL) v4: recent updates and new developments. Nucleic Acids Res. 47, 256–259. doi: 10.1093/nar/gkz239 PMC6602468 30931475

[B37] Li Y.-H. Reif J. C. Jackson S. A. Ma Y.-S. Chang R.-Z. Qiu L.-J. (2014). Detecting SNPs underlying domestication-related traits in soybean. BMC Plant Biol. 14, 1–8. doi: 10.1186/s12870-014-0251-1 PMC4180965 25258093

[B38] Magbagbeola J. Adetoso J. Owolabi O. (2010). Neglected and underutilized species (NUS): a panacea for community focused development to poverty alleviation/poverty reduction in Nigeria. J. Economics Int. Finance 2, 208–211. doi: 10.5897/JEIF.9000082

[B39] Olango T. M. Tesfaye B. Pagnotta M. A. Pè M. E. Catellani M. (2015). Development of SSR markers and genetic diversity analysis in enset (*Ensete ventricosum* (Welw.) Cheesman), an orphan food security crop from Southern Ethiopia. BMC Genet. 16, 98. doi: 10.1186/s12863-015-0250-8 26243662 PMC4524394

[B40] Papa R. Bellucci E. Rossi M. Leonardi S. Rau D. Gepts P.. (2007). Tagging the signatures of domestication in common bean (*Phaseolus vulgaris*) by means of pooled DNA samples. Ann. Bot. 100, 1039–1051. doi: 10.1093/aob/mcm151 17673468 PMC2759209

[B41] Peterson B. K. Weber J. N. Kay E. H. Fisher H. S. Hoekstra H. E. (2012). Double digest RADseq: an inexpensive method for *de novo* SNP discovery and genotyping in model and non-model species. PloS One 7, e37135. doi: 10.1371/journal.pone.0037135 22675423 PMC3365034

[B42] Raj A. Stephens M. Pritchard J. K. (2014). ) fastSTRUCTURE: variational inference of population structure in large SNP data sets. Genetics 197, 573–589. doi: 10.1534/genetics.114.164350 24700103 PMC4063916

[B43] Rochette N. C. Rivera-Colón A. G. Catchen J. M. (2019). Stacks 2: Analytical methods for paired-end sequencing improve RADseq-based population genomics. Mol. Ecol. 28, 4737–4754. doi: 10.1111/mec.15253 31550391

[B44] Rogstad S. H. (1992). Saturated NaCl-CTAB solution as a means of field preservation of leaves for DNA analyses. *Taxon* . 41(1), 701–708. doi: 10.2307/1222395

[B45] Rousset F. (2002). Inbreeding and relatedness coefficients: what do they measure? Heredity 88, 371–380. doi: 10.1038/sj/hdy/6800065 11986874

[B46] Sardos J. Breton C. Perrier X. Van den Houwe I. Carpentier S. Paofa J.. (2022). Hybridization, missing wild ancestors and the domestication of cultivated diploid bananas. Front. Plant Sci. 3668. doi: 10.3389/fpls.2022.969220 PMC9586208 36275535

[B47] Schaal B. Hayworth D. Olsen K. M. Rauscher J. Smith W. (1998). Phylogeographic studies in plants: problems and prospects. Mol. Ecol. 7, 465–474. doi: 10.1046/j.1365-294x.1998.00318.x

[B48] Schaller G. E. Bishopp A. Kieber J. J. (2015). The yin-yang of hormones: cytokinin and auxin interactions in plant development. Plant Cell 27, 44–63. doi: 10.1105/tpc.114.133595 25604447 PMC4330578

[B49] Schumacher K. Schmitt T. Rossberg M. Schmitz G. Theres K. (1999). The Lateral suppressor (Ls) gene of tomato encodes a new member of the VHIID protein family. Proc. Natl. Acad. Sci. 96, 290–295. doi: 10.1073/pnas.96.1.290 9874811 PMC15132

[B50] Shigeta M. (1990). 'Folk In-Situ Conservation of Ensete [*Ensete ventricosum* (Welw.) E.E. Cheesman]: Toward the Interpretation of Indigenous Agricultural Science of the Ari, Sowthwestern Ethiopia. African Study Monographs 10(3): 93–107. doi: 10.11501/3086425

[B51] Shigeta M. (1992). The ethnobotanical study of ensete (*Ensete ventricosum*) in the southwestern Ethiopia Kyoto University]. Japan. doi: 10.11501/3086425

[B52] Sim S. Robbins M. Van Deynze A. Michel A. Francis D. (2011). Population structure and genetic differentiation associated with breeding history and selection in tomato (*Solanum lycopersicum* L.). Heredity 106, 927–935. doi: 10.1038/hdy.2010.139 21081965 PMC3186243

[B53] Tsykun T. Rellstab C. Dutech C. Sipos G. Prospero S. (2017). Comparative assessment of SSR and SNP markers for inferring the population genetic structure of the common fungus Armillaria cepistipes. Heredity 119, 371–380. doi: 10.1038/hdy.2017.48 28813039 PMC5637368

[B54] Weir B. S. Cockerham C. C. (1984). Estimating F-statistics for the analysis of population structure. Evolution 38, 1358–1370. doi: 10.1111/j.1558-5646.1984.tb05657.x 28563791

[B55] Werner T. Motyka V. Laucou V. Smets R. Van Onckelen H. Schmülling T. (2003). Cytokinin-deficient transgenic *Arabidopsis* plants show multiple developmental alterations indicating opposite functions of cytokinins in the regulation of shoot and root meristem activity. Plant Cell 15, 2532–2550. doi: 10.1105/tpc.014928 14555694 PMC280559

[B56] White O. W. Biswas M. K. Abebe W. M. Dussert Y. Kebede F. Nichols R. A.. (2023). Maintenance and expansion of genetic and trait variation following domestication in a clonal crop. Mol. Ecol. 32, 4165–4180. doi: 10.1111/mec.17033 37264989

[B57] Yemataw Z. Muzemil S. Ambachew D. Tripathi L. Tesfaye K. Chala A.. (2018). Genome sequence data from 17 accessions of *Ensete ventricosum*, a staple food crop for millions in Ethiopia. Data Brief 18, 285–293. doi: 10.1016/j.dib.2018.03.026 29896517 PMC5996239

[B58] Zeven A. C. (1998). Landraces: A review of definitions and classifications. Euphytica 104, 127–139. doi: 10.1023/a:1018683119237

[B59] Zhang X. Yan F. Tang Y. Yuan Y. Deng W. Li Z. (2015). Auxin response gene SlARF3 plays multiple roles in tomato development and is involved in the formation of epidermal cells and trichomes. Plant Cell Physiol. 56, 2110–2124. doi: 10.1093/pcp/pcv136 26412778

