# Supplementary figures and images for: Limited genetic diversity found among genotypes of the Entada landrace (Ensete ventricosum, (Welw.) Chessman) from Ethiopia
Source: Front Plant Sci. 2024 Sep 9;15:1336461. doi: 10.3389/fpls.2024.1336461 (PMC11416936; doi:10.3389/fpls.2024.1336461)

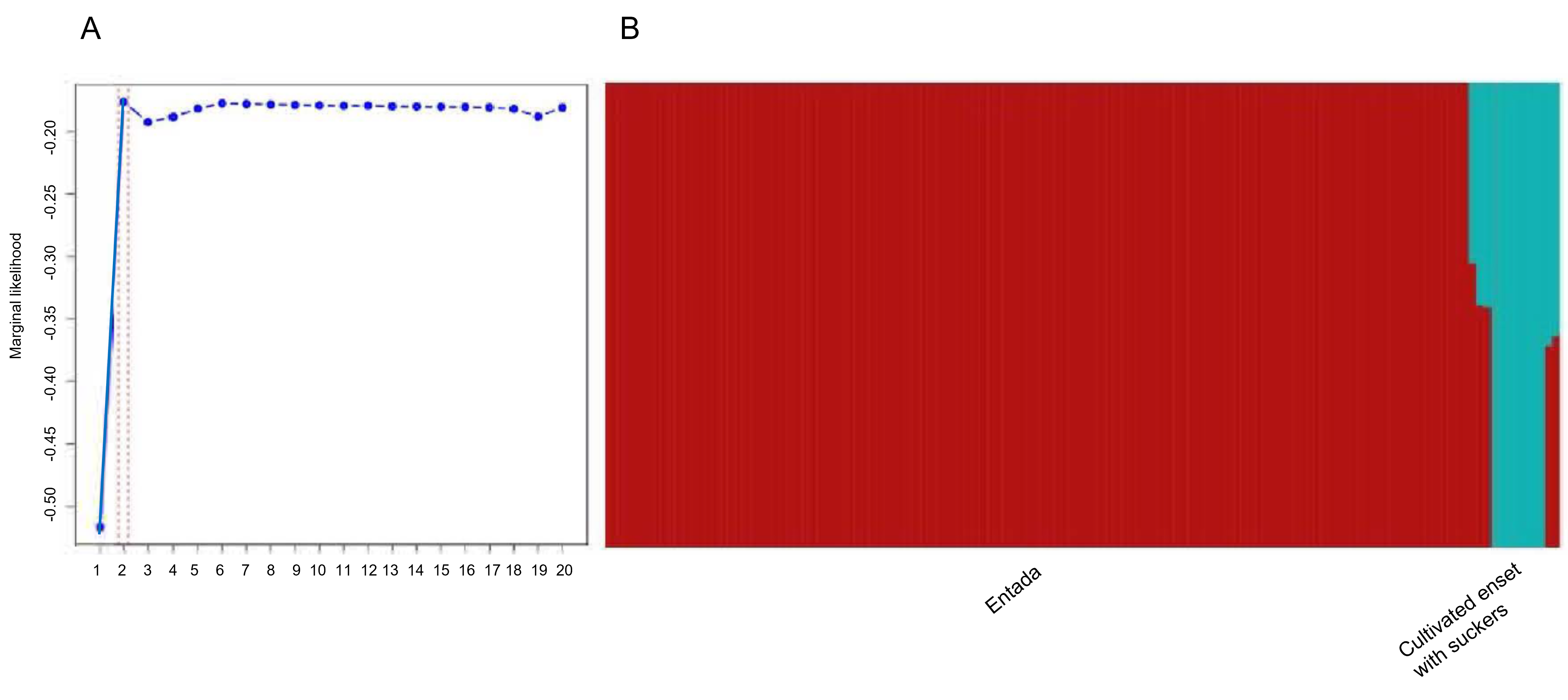

Supplement: Supplementary Figure 1 — Population structure of the 117 Entada genotypes and the 12 cultivated enset with suckers. (A) The model complexity that maximizes likelihood is 2. The highest peak shows the best K = 2. (B) Population structure based on fastSTRUCTURE output resulting in K = 2 being the most likely number of genetic clusters. Entada genotypes = red; cultivated enset with suckers = aqua. [file Image1.tif]
