# Supplementary material for: Limited genetic diversity found among genotypes of the Entada landrace (Ensete ventricosum, (Welw.) Chessman) from Ethiopia
Source: Front Plant Sci. 2024 Sep 9;15:1336461. doi: 10.3389/fpls.2024.1336461 (PMC11416936; doi:10.3389/fpls.2024.1336461)
Supplement: Supplementary file 2 [file Table1.pdf]

Supplementary Table 1. Geographical locations of 117 Entada (*Ensete ventricosum* (Welw.) Cheesman) genotypes used for analysis of genetic diversity of the Entada landrace.

Genotype ID	Region	Geographical coordinates (DMS)	
238	South Ari	05 59 00.1	036 34 36.7
239	South Ari	05 58 53.4	036 34 26.4
240	South Ari	05 58 50.0	036 34 21.4
241	South Ari	05 58 28.8	036 34 22.9
242	South Ari	05 58 29.0	036 34 18.8
243	South Ari	05 58 45.5	036 34 14.8
244	South Ari	05 55 40.8	036 34 54.3
246	South Ari	05 47 17.9	036 33 19.1
247	South Ari	05 47 13.4	036 37 46.9
248	South Ari	N/A	N/A
249	South Ari	N/A	N/A
254	Sidama	07 07 06.1	038 30 19.9
255	Sidama	07 07 06.1	038 30 19.9
256	Sidama	07 07 06.1	038 30 19.9
260	Sidama	07 07 06.1	038 30 19.9
261	Sidama	07 07 06.1	038 30 19.9
263	Sidama	07 07 06.1	038 30 19.9
264	Sidama	07 07 06.1	038 30 19.9
265	Sidama	07 07 06.1	038 30 19.9
266	Sidama	07 07 06.1	038 30 19.9
267	Sidama	07 07 06.1	038 30 19.9
268	Sidama	07 07 06.1	038 30 19.9
269	Sidama	07 07 06.1	038 30 19.9
270	Sidama	07 07 06.1	038 30 19.9
271	South Ari	N/A	N/A
272	South Ari	05 47 17.8	036 33 09.8
273	South Ari	05 47 10.3	036 33 11.0
274	South Ari	05 47 13.5	036 33 06.5
275	South Ari	05 47 13.5	036 33 06.5
276	South Ari	05 47 19.7	036 33 02.6
278	South Ari	05 48 49.6	036 32 59.4
279	South Ari	05 47 34.9	036 33 49.1
280	South Ari	05 51 15.7	036 32 56.3
281	South Ari	05 52 15.8	036 32 51.5
282	South Ari	05 51 15.4	036 33 08.8
283	South Ari	05 51 06.6	036 33 02.4
284	South Ari	05 51 06.4	036 33 02.4
285	South Ari	05 51 06.4	036 33 02.4

Genotype ID	Region	Geographical coordinates (DMS)	
286	South Ari	05 50 56.7	036 33 01.6
287	South Ari	05 50 56.7	036 33 02.5
288	South Ari	05 50 55.0	036 33 02.8
289	South Ari	05 57 37.4	036 33 06.7
290	South Ari	05 57 31.8	036 33 05.3
291	South Ari	05 57 35.2	036 33 05.6
292	South Ari	05 57 15.2	036 33 10.1
293	South Ari	05 57 31.4	036 33 07.6
294	South Ari	05 57 31.4	036 3307.6
295	South Ari	05 57 55.7	036 33 09.1
296	South Ari	05 58 29.0	036 34 18.8
297	South Ari	05 58 27.7	036 34 18.5
298	South Ari	05 58 46.8	036 34 18.7
299	South Ari	05 58 45.3	036 34 05.8
300	South Ari	05 58 47.9	036 36 21.8
301	South Ari	05 58 52.7	036 34 24.8
302	South Ari	05 59 18.7	036 34 45.5
303	South Ari	05 59 17.1	036 34 56.5
304	South Ari	05 59 19.6	036 35 08.5
305	South Ari	05 59 19.5	036 35 09.0
306	South Ari	05 59 19.2	036 35 01.7
307	South Ari	05 58 27.6	036 34 57.1
309	South Ari	05 58 08.9	036 35 21.9
310	South Ari	05 56 25.6	036 35 07.9
311	South Ari	05 56 22.4	036 35 06.2
312	South Ari	05 58 11.3	036 35 14.9
313	South Ari	06 03 37.4	036 32 14.1
314	South Ari	06 03 34.8	036 32 18.6
315	South Ari	06 04 37.8	036 32 15.1
316	South Ari	06 03 34.8	036 32 18.6
317	South Ari	06 03 34.8	036 32 18.6
318	South Ari	N/A	N/A
319	South Ari	06 05 56.8	036 32 47.0
320	South Ari	06 05 54.7	036 32 47.5
322	South Ari	06 06 18.4	036 32 40.9
323	South Ari	06 06 35.3	036 32 34.3
324	South Ari	06 05 03.2	036 32 25.2
325	South Ari	06 06 35.3	036 32 43.3
326	South Ari	06 03 58.3	036 32 21.5
327	South Ari	06 03 44.5	036 32 17.3
328	South Ari	06 03 15.5	036 31 59.2

Genotype ID	Region	Geographical coordinates (DMS)	
329	South Ari	06 02 51.1	036 32 06.2
330	South Ari	06 02 34.7	036 32 10.9
331	South Ari	06 02 21.8	036 32 09.8
332	South Ari	06 02 16.1	036 32 07.1
333	South Ari	06 01 50.7	036 32 19.7
334	South Ari	06 01 37.0	036 32 11.5
335	South Ari	06 01 36.5	036 32 10.4
336	South Ari	06 01 55.9	036 32 17.4
337	South Ari	06 02 36.1	036 32 11.2
338	South Ari	06 02 36.1	036 32 11.2
339	South Ari	N/A	N/A
340	South Ari	N/A	N/A
341	South Ari	N/A	N/A
342	South Ari	N/A	N/A
343	South Ari	N/A	N/A
344	South Ari	N/A	N/A
345	South Ari	N/A	N/A
346	South Ari	N/A	N/A
347	South Ari	N/A	N/A
348	South Ari	N/A	N/A
349	South Ari	N/A	N/A
350	North Ari	06 12 55.3	036 38 11.2
351	North Ari	06 12 36.1	036 38 07.9
352	North Ari	06 12 55.3	036 38 11.2
353	North Ari	N/A	N/A
354	North Ari	N/A	N/A
355	North Ari	06 12 55.3	036 38 11.2
356	North Ari	N/A	N/A
357	North Ari	N/A	N/A
358	North Ari	06 13 02.6	036 38 06.5
359	North Ari	06 13 02.6	036 38 06.5
360	North Ari	06 13 02.6	036 38 06.5
361	North Ari	06 13 02.6	036 38 06.5
362	North Ari	06 14 08.7	036 37 18.9
363	North Ari	06 13 59.9	036 37 30.0
364	North Ari	06 13 59.9	036 37 30.0
365	North Ari	N/A	N/A
367	North Ari	N/A	N/A

N/A= coordinates not available.
