# Supplementary material for: Limited genetic diversity found among genotypes of the Entada landrace (Ensete ventricosum, (Welw.) Chessman) from Ethiopia
Source: Front Plant Sci. 2024 Sep 9;15:1336461. doi: 10.3389/fpls.2024.1336461 (PMC11416936; doi:10.3389/fpls.2024.1336461)
Supplement: Supplementary file 3 [file Table2.pdf]

Supplementary Table 2. Unique P1-EcoRI barcode adapters ligated to DNA fragments from each sample.

Name	OligoSequence
GCATG_EcoRI_P1.1	ACACTCTTTCCCTACACGACGCTCTTCCGATCTGCATG
AACCA_EcoRI_P1.1	ACACTCTTTCCCTACACGACGCTCTTCCGATCTAACCA
CGATC_EcoRI_P1.1	ACACTCTTTCCCTACACGACGCTCTTCCGATCTCGATC
TCGAT_EcoRI_P1.1	ACACTCTTTCCCTACACGACGCTCTTCCGATCTTCGAT
TGCAT_EcoRI_P1.1	ACACTCTTTCCCTACACGACGCTCTTCCGATCTTGCAT
CAACC_EcoRI_P1.1	ACACTCTTTCCCTACACGACGCTCTTCCGATCTCAACC
GGTTG_EcoRI_P1.1	ACACTCTTTCCCTACACGACGCTCTTCCGATCTGGTTG
AAGGA_EcoRI_P1.1	ACACTCTTTCCCTACACGACGCTCTTCCGATCTAAGGA
AGCTA_EcoRI_P1.1	ACACTCTTTCCCTACACGACGCTCTTCCGATCTAGCTA
ACACA_EcoRI_P1.1	ACACTCTTTCCCTACACGACGCTCTTCCGATCTACACA
AATTA_EcoRI_P1.1	ACACTCTTTCCCTACACGACGCTCTTCCGATCTAATTA
ACGGT_EcoRI_P1.1	ACACTCTTTCCCTACACGACGCTCTTCCGATCTACGGT
ACTGG_EcoRI_P1.1	ACACTCTTTCCCTACACGACGCTCTTCCGATCTACTGG
ACTTC_EcoRI_P1.1	ACACTCTTTCCCTACACGACGCTCTTCCGATCTACTTC
ATACG_EcoRI_P1.1	ACACTCTTTCCCTACACGACGCTCTTCCGATCTATACG
ATGAG_EcoRI_P1.1	ACACTCTTTCCCTACACGACGCTCTTCCGATCTATGAG
ATTAC_EcoRI_P1.1	ACACTCTTTCCCTACACGACGCTCTTCCGATCTATTAC
CATAT_EcoRI_P1.1	ACACTCTTTCCCTACACGACGCTCTTCCGATCTCATAT
CGAAT_EcoRI_P1.1	ACACTCTTTCCCTACACGACGCTCTTCCGATCTCGAAT
CGGCT_EcoRI_P1.1	ACACTCTTTCCCTACACGACGCTCTTCCGATCTCGGCT
CGGTA_EcoRI_P1.1	ACACTCTTTCCCTACACGACGCTCTTCCGATCTCGGTA
CGTAC_EcoRI_P1.1	ACACTCTTTCCCTACACGACGCTCTTCCGATCTCGTAC
CGTCG_EcoRI_P1.1	ACACTCTTTCCCTACACGACGCTCTTCCGATCTCGTCG
CTGAT_EcoRI_P1.1	ACACTCTTTCCCTACACGACGCTCTTCCGATCTCTGAT
CTGCG_EcoRI_P1.1	ACACTCTTTCCCTACACGACGCTCTTCCGATCTCTGCG
CTGTC_EcoRI_P1.1	ACACTCTTTCCCTACACGACGCTCTTCCGATCTCTGTC
CTTGG_EcoRI_P1.1	ACACTCTTTCCCTACACGACGCTCTTCCGATCTCTTGG
GACAC_EcoRI_P1.1	ACACTCTTTCCCTACACGACGCTCTTCCGATCTGACAC
GAGAT_EcoRI_P1.1	ACACTCTTTCCCTACACGACGCTCTTCCGATCTGAGAT
GAGTC_EcoRI_P1.1	ACACTCTTTCCCTACACGACGCTCTTCCGATCTGAGTC
GCCGT_EcoRI_P1.1	ACACTCTTTCCCTACACGACGCTCTTCCGATCTGCCGT
GCTGA_EcoRI_P1.1	ACACTCTTTCCCTACACGACGCTCTTCCGATCTGCTGA
GGATA_EcoRI_P1.1	ACACTCTTTCCCTACACGACGCTCTTCCGATCTGGATA
GGCCA_EcoRI_P1.1	ACACTCTTTCCCTACACGACGCTCTTCCGATCTGGCCA
GGCTC_EcoRI_P1.1	ACACTCTTTCCCTACACGACGCTCTTCCGATCTGGCTC
GTAGT_EcoRI_P1.1	ACACTCTTTCCCTACACGACGCTCTTCCGATCTGTAGT
GTCCG_EcoRI_P1.1	ACACTCTTTCCCTACACGACGCTCTTCCGATCTGTCCG
GTCGA_EcoRI_P1.1	ACACTCTTTCCCTACACGACGCTCTTCCGATCTGTCGA
TACCG_EcoRI_P1.1	ACACTCTTTCCCTACACGACGCTCTTCCGATCTTACCG
TACGT_EcoRI_P1.1	ACACTCTTTCCCTACACGACGCTCTTCCGATCTTACGT
TAGTA_EcoRI_P1.1	ACACTCTTTCCCTACACGACGCTCTTCCGATCTTAGTA
TATAC_EcoRI_P1.1	ACACTCTTTCCCTACACGACGCTCTTCCGATCTTATAC
TCACG_EcoRI_P1.1	ACACTCTTTCCCTACACGACGCTCTTCCGATCTTCACG
TCAGT_EcoRI_P1.1	ACACTCTTTCCCTACACGACGCTCTTCCGATCTTCAGT
TCCGG_EcoRI_P1.1	ACACTCTTTCCCTACACGACGCTCTTCCGATCTTCCGG
TCTGC_EcoRI_P1.1	ACACTCTTTCCCTACACGACGCTCTTCCGATCTTCTGC
TGGAA_EcoRI_P1.1	ACACTCTTTCCCTACACGACGCTCTTCCGATCTTGGAA

Supplementary Table 2. Continued …

TTACC_EcoRI_P1.1	ACACTCTTTCCCTACACGACGCTCTTCCGATCTTTACC
GCATG_EcoRI_P1.2	/5Phos/AATTCATGCAGATCGGAAGAGCGTCGTGTAGGGAAAGAGTGT
AACCA_EcoRI_P1.2	/5Phos/AATTTGGTTAGATCGGAAGAGCGTCGTGTAGGGAAAGAGTGT
CGATC_EcoRI_P1.2	/5Phos/AATTGATCGAGATCGGAAGAGCGTCGTGTAGGGAAAGAGTGT
TCGAT_EcoRI_P1.2	/5Phos/AATTATCGAAGATCGGAAGAGCGTCGTGTAGGGAAAGAGTGT
TGCAT_EcoRI_P1.2	/5Phos/AATTATGCAAGATCGGAAGAGCGTCGTGTAGGGAAAGAGTGT
CAACC_EcoRI_P1.2	/5Phos/AATTGGTTGAGATCGGAAGAGCGTCGTGTAGGGAAAGAGTGT
GGTTG_EcoRI_P1.2	/5Phos/AATTCAACCAGATCGGAAGAGCGTCGTGTAGGGAAAGAGTGT
AAGGA_EcoRI_P1.2	/5Phos/AATTTCCTTAGATCGGAAGAGCGTCGTGTAGGGAAAGAGTGT
AGCTA_EcoRI_P1.2	/5Phos/AATTTAGCTAGATCGGAAGAGCGTCGTGTAGGGAAAGAGTGT
ACACA_EcoRI_P1.2	/5Phos/AATTTGTGTAGATCGGAAGAGCGTCGTGTAGGGAAAGAGTGT
AATTA_EcoRI_P1.2	/5Phos/AATTTAATTAGATCGGAAGAGCGTCGTGTAGGGAAAGAGTGT
ACGGT_EcoRI_P1.2	/5Phos/AATTACCGTAGATCGGAAGAGCGTCGTGTAGGGAAAGAGTGT
ACTGG_EcoRI_P1.2	/5Phos/AATTCCAGTAGATCGGAAGAGCGTCGTGTAGGGAAAGAGTGT
ACTTC_EcoRI_P1.2	/5Phos/AATTGAAGTAGATCGGAAGAGCGTCGTGTAGGGAAAGAGTGT
ATACG_EcoRI_P1.2	/5Phos/AATTCGTATAGATCGGAAGAGCGTCGTGTAGGGAAAGAGTGT
ATGAG_EcoRI_P1.2	/5Phos/AATTCTCATAGATCGGAAGAGCGTCGTGTAGGGAAAGAGTGT
ATTAC_EcoRI_P1.2	/5Phos/AATTGTAATAGATCGGAAGAGCGTCGTGTAGGGAAAGAGTGT
CATAT_EcoRI_P1.2	/5Phos/AATTATATGAGATCGGAAGAGCGTCGTGTAGGGAAAGAGTGT
CGAAT_EcoRI_P1.2	/5Phos/AATTATTCGAGATCGGAAGAGCGTCGTGTAGGGAAAGAGTGT
CGGCT_EcoRI_P1.2	/5Phos/AATTAGCCGAGATCGGAAGAGCGTCGTGTAGGGAAAGAGTGT
CGGTA_EcoRI_P1.2	/5Phos/AATTTACCGAGATCGGAAGAGCGTCGTGTAGGGAAAGAGTGT
CGTAC_EcoRI_P1.2	/5Phos/AATTGTACGAGATCGGAAGAGCGTCGTGTAGGGAAAGAGTGT
CGTCG_EcoRI_P1.2	/5Phos/AATTCGACGAGATCGGAAGAGCGTCGTGTAGGGAAAGAGTGT
CTGAT_EcoRI_P1.2	/5Phos/AATTATCAGAGATCGGAAGAGCGTCGTGTAGGGAAAGAGTGT
CTGCG_EcoRI_P1.2	/5Phos/AATTCGCAGAGATCGGAAGAGCGTCGTGTAGGGAAAGAGTGT
CTGTC_EcoRI_P1.2	/5Phos/AATTGACAGAGATCGGAAGAGCGTCGTGTAGGGAAAGAGTGT
CTTGG_EcoRI_P1.2	/5Phos/AATTCCAAGAGATCGGAAGAGCGTCGTGTAGGGAAAGAGTGT
GACAC_EcoRI_P1.2	/5Phos/AATTGTGTCAGATCGGAAGAGCGTCGTGTAGGGAAAGAGTGT
GAGAT_EcoRI_P1.2	/5Phos/AATTATCTCAGATCGGAAGAGCGTCGTGTAGGGAAAGAGTGT
GAGTC_EcoRI_P1.2	/5Phos/AATTGACTCAGATCGGAAGAGCGTCGTGTAGGGAAAGAGTGT
GCCGT_EcoRI_P1.2	/5Phos/AATTACGGCAGATCGGAAGAGCGTCGTGTAGGGAAAGAGTGT
GCTGA_EcoRI_P1.2	/5Phos/AATTTCAGCAGATCGGAAGAGCGTCGTGTAGGGAAAGAGTGT
GGATA_EcoRI_P1.2	/5Phos/AATTTATCCAGATCGGAAGAGCGTCGTGTAGGGAAAGAGTGT
GGCCA_EcoRI_P1.2	/5Phos/AATTTGGCCAGATCGGAAGAGCGTCGTGTAGGGAAAGAGTGT
GGCTC_EcoRI_P1.2	/5Phos/AATTGAGCCAGATCGGAAGAGCGTCGTGTAGGGAAAGAGTGT
GTAGT_EcoRI_P1.2	/5Phos/AATTACTACAGATCGGAAGAGCGTCGTGTAGGGAAAGAGTGT
GTCCG_EcoRI_P1.2	/5Phos/AATTCGGACAGATCGGAAGAGCGTCGTGTAGGGAAAGAGTGT
GTCGA_EcoRI_P1.2	/5Phos/AATTTCGACAGATCGGAAGAGCGTCGTGTAGGGAAAGAGTGT
TACCG_EcoRI_P1.2	/5Phos/AATTCGGTAAGATCGGAAGAGCGTCGTGTAGGGAAAGAGTGT
TACGT_EcoRI_P1.2	/5Phos/AATTACGTAAGATCGGAAGAGCGTCGTGTAGGGAAAGAGTGT
TAGTA_EcoRI_P1.2	/5Phos/AATTTACTAAGATCGGAAGAGCGTCGTGTAGGGAAAGAGTGT
TATAC_EcoRI_P1.2	/5Phos/AATTGTATAAGATCGGAAGAGCGTCGTGTAGGGAAAGAGTGT
TCACG_EcoRI_P1.2	/5Phos/AATTCGTGAAGATCGGAAGAGCGTCGTGTAGGGAAAGAGTGT
TCAGT_EcoRI_P1.2	/5Phos/AATTACTGAAGATCGGAAGAGCGTCGTGTAGGGAAAGAGTGT
TCCGG_EcoRI_P1.2	/5Phos/AATTCCGGAAGATCGGAAGAGCGTCGTGTAGGGAAAGAGTGT
TCTGC_EcoRI_P1.2	/5Phos/AATTGCAGAAGATCGGAAGAGCGTCGTGTAGGGAAAGAGTGT
TGGAA_EcoRI_P1.2	/5Phos/AATTTTCCAAGATCGGAAGAGCGTCGTGTAGGGAAAGAGTGT
TTACC_EcoRI_P1.2	/5Phos/AATTGGTAAAGATCGGAAGAGCGTCGTGTAGGGAAAGAGTGT
