# Supplementary material for: Limited genetic diversity found among genotypes of the Entada landrace (Ensete ventricosum, (Welw.) Chessman) from Ethiopia
Source: Front Plant Sci. 2024 Sep 9;15:1336461. doi: 10.3389/fpls.2024.1336461 (PMC11416936; doi:10.3389/fpls.2024.1336461)
Supplement: Supplementary file 4 [file Table3.pdf]

Supplementary Table 3. Common P2-Mspl barcode adapters ligated to DNA fragments from all samples.

Name	OligoSequence
MspI_P2.1	GTGACTGGAGTTCAGACGTGTGCTCTTCCGATCT
MspI_P2.2	/5Phos/CGAGATCGGAAGAGCGAGAACAA
