# Supplementary material for: Limited genetic diversity found among genotypes of the Entada landrace (Ensete ventricosum, (Welw.) Chessman) from Ethiopia
Source: Front Plant Sci. 2024 Sep 9;15:1336461. doi: 10.3389/fpls.2024.1336461 (PMC11416936; doi:10.3389/fpls.2024.1336461)
Supplement: Supplementary file 5 [file Table4.pdf]

Supplementary Table 4. PCR primers for amplification of libraries and tagging of the samples.

Name	OligoSequence
PCR1	AATGATACGGCGACCACCGAGATCTACACTCTTTCCCTACACGACG
PCR2_Idx_6_GCCAAT	CAAGCAGAAGACGGCATACGAGATATTGGCGTGACTGGAGTTCAGACGTGTGC
PCR2_Idx_12_CTTGTA	CAAGCAGAAGACGGCATACGAGATTACAAGGTGACTGGAGTTCAGACGTGTGC
